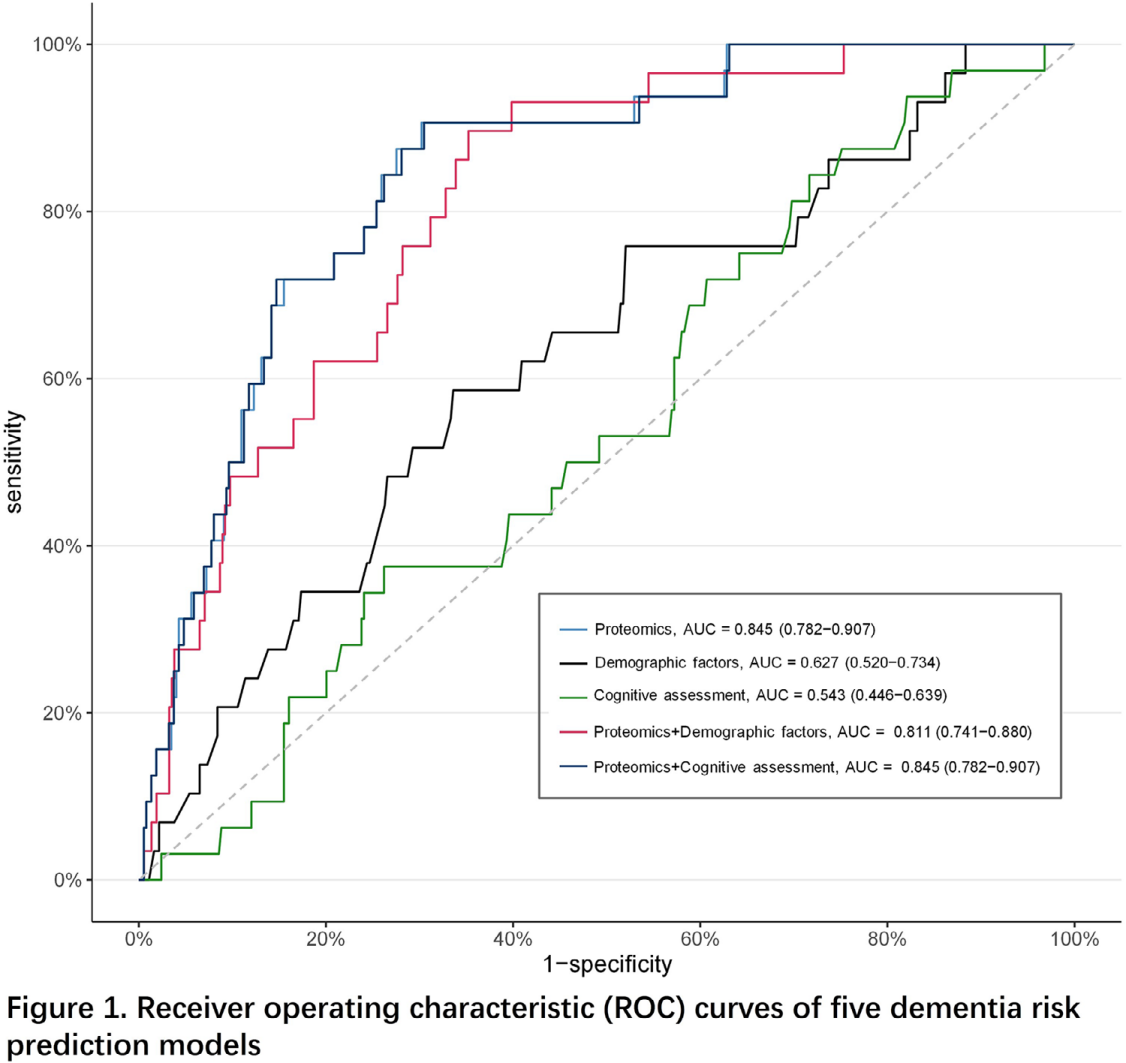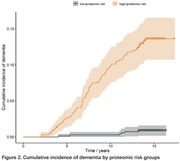# Proteomic Dementia Risk Assessment in Chronic Kidney Disease

**DOI:** 10.1002/alz.092124

**Published:** 2025-01-09

**Authors:** Bote Zhao, Zhibo Wang, Yuye Ning, Jianping Jia

**Affiliations:** ^1^ Xuanwu Hospital, Capital Medical University, Beijing China; ^2^ Xuanwu Hospital, Capital Medical University, Beijing, Beijing China; ^3^ Key Laboratory of Neurodegenerative Diseases, Ministry of Education, Beijing China; ^4^ Beijing Key Laboratory of Geriatric Cognitive Disorders, Beijing China; ^5^ Center of Alzheimer’s Disease, Beijing Institute for Brain Disorders, Beijing China; ^6^ Innovation Center for Neurological Disorders, Xuanwu Hospital, Capital Medical University, Beijing, Beijing China; ^7^ National Clinical Research Center for Geriatric Disorders, Beijing China

## Abstract

**Background:**

Chronic kidney disease (CKD) has relatively high prevalence and independently increases dementia risk. Currently, there is a lack of high‐performance dementia prediction tools designed for the CKD population in clinical practice. Through proteomics discovery, this study aimed to discover more efficient dementia risk models in CKD patients.

**Method:**

This prospective cohort study utilized data from the UK Biobank, encompassing baseline assessments conducted between 2006 and 2010, with follow‐up extended until September 1, 2023. We included 1355 CKD patients, who were free of dementia at baseline and had available Olink blood proteomics data (approximately 3000 proteins). Feature selection was executed using a least absolute shrinkage and selection operator (LASSO) regression. Generalized Linear Models were employed to derive a proteomic risk model for dementia risk in CKD patients. The model was trained in a tested set of 949 CKD patients and validated in a set of 406 individuals. Mendelian randomization was employed to investigate the causal link between potential proteins and dementia.

**Result:**

The proteomic risk model, comprised of 17 proteins, demonstrated superior performance compared to conventional clinical tools for dementia risk assessment, including demographic factors (age, sex, education year, APOE ε4 status) and cognitive assessments. During validation, the proteomic model achieved a receiver operating characteristic (ROC) area under the curve (AUC) value of 0.845, significantly surpassing the 0.543 to 0.627 range observed for conventional clinical models. The proteomic plus conventional assessment models did not obtain higher AUC values (ranging from 0.811 to 0.845) (Figure 1). After stratifying participants into two proteomic risk groups based on the median proteomic predicted risk value, the high‐proteomic risk group exhibited 15.14 times increase in the risk of dementia compared to the low‐ proteomic risk group (HR= 15.14, 95%CI: 6.62 ‐ 34.66) (Figure 2). Mendelian randomization analyses indicated a causal link between several proteins and dementia.

**Conclusion:**

Based on the UK Biobank database, a proteomic risk model for dementia outperformed conventional clinical risk models in CKD patients. This plasma‐derived proteomic model is supposed to have great generalization potential, attributed to the accessibility of blood samples, thereby offering valuable guidance for dementia prevention in the CKD population.